# Equine penile squamous cell carcinoma: expression of biomarker proteins and EcPV2

**DOI:** 10.1038/s41598-020-64014-3

**Published:** 2020-05-12

**Authors:** Callum Arthurs, Alejandro Suarez-Bonnet, Claire Willis, Boyu Xie, Natalie Machulla, Tim S. Mair, Kevin Cao, Michael Millar, Christopher Thrasivoulou, Simon L. Priestnall, Aamir Ahmed

**Affiliations:** 1 Prostate Cancer Research Centre at the Centre for Stem Cells and Regenerative Medicine, King’s College London, London, United Kingdom; 2 Department of Pathobiology and Population Sciences, Royal Veterinary College, Hertfordshire, UK; 3 Bell Equine Veterinary Clinic, Maidstone, UK; 4 Queen’s Medical Research Institute, University of Edinburgh, Edinburgh, United Kingdom; 5 Research Department of Cell and Developmental Biology, The Centre for Cell and Molecular Dynamics, Rockefeller Building, University College London, London, United Kingdom

**Keywords:** Tumour biomarkers, Proteomics

## Abstract

Equine penile squamous cell carcinoma (EpSCC) is a relatively common cutaneous neoplasm with a poor prognosis. In this study, we aimed to determine the protein expression and colocalisation of FRA1, c-Myc, Cyclin D1, and MMP7 in normal (NT), tumour (T), hyperplastic epidermis and/or squamous papilloma (Hyp/Pap), poorly-differentiated (PDSCC), or well-differentiated (WDSCC) EpSCC using a tissue array approach. Further objectives were to correlate protein expression to (i) levels of inflammation, using a convolutional neural network (ii) equine papillomavirus 2 (EcPV2) infection, detected using PCR amplification. We found an increase in expression of FRA1 in EpSCC compared to NT samples. c-Myc expression was higher in Hyp/Pap and WDSCC but not PDSCC whereas MMP7 was reduced in WDSCC compared with NT. There was a significant increase in the global intersection coefficient (GIC) of FRA1 with MMP7, c-Myc, and Cyclin D1 in EpSCC. Conversely, GIC for MMP7 with c-Myc was reduced in EpSCC tissue. Inflammation was positively associated with EcPV2 infection in both NT and EpSCC but not Hyp/Pap. Changes in protein expression could be correlated with EcPV2 for Cyclin D1 and c-Myc. Our results evaluate novel biomarkers of EpSCC and a putative correlation between the expression of biomarkers, EcPV2 infection and inflammation.

## Introduction

Equine penile squamous cell carcinoma (EpSCC) is a cutaneous neoplasm with a poor prognosis that often results in euthanasia due to late presentation, treatment difficulties and deterioration. EpSCC are often seen with precancerous pink to yellow plaques and genital papillomas. The lesion is seen mostly at the end of the second and beginning of the third decade of life^1^. The term penile intraepithelial neoplasia (PIN) used in humans may also be applied to these lesions.

After sarcoids, squamous cell carcinomas are considered the most common equine neoplasm^1–3^. Around one tenth of all equine neoplasms are diagnosed in the penis, vulva and ocular adnexa^4,5^ of which EpSCC is the most common. Incidence rates of EpSCC, reported more in ponies compared to horses^6^, vary and no specific breed predilection has been ascertained^6^. The recorded incidence rates for EpSCCs are between 50–80% of all external genital neoplasms, however one report recorded that EpSCC made up around a fifth of all diagnosed equine cancers in a single UK laboratory over a 29-year period, with the incidence of cutaneous equine tumours also varying by region^6^.

The possible causes of EpSCC are suggested to be smegma accumulation, ultraviolet light overexposure, chronic irritation and balanoposthitis^7^. Chronic inflammation is a known risk factor for cancer development^8^. It is also thought that a majority of solid tumours are infiltrated with immune and inflammatory cells^9^. The link between human papilloma virus (HPV), cervical cancer^10^ and chronic inflammation is known^8^. There is evidence to suggest that equine cancers may be initiated, in part, by papillomavirus infection analogous to human cervical and penile cancer^11^. These suggest an inflammation^7^ and equine papillomavirus 2 (EcPV2) infection driven oncogenesis^7,12^, similar to the sexually-transmitted infection (STI) model proposed in human cervical cancer^13^.

A 2007 study investigated the presence of EcPV1 in a selection of equine papilloma, aural plaque, sarcoid and normal tissue samples with results suggesting that >50% of cutaneous papilloma samples tested positive for EcPV1 but the virus was not found in the small number of genital samples that were tested^14^. In other studies^7,15,16^, EcPV2, a papillomavirus from a separate genus to EcPV1^17^, has been suggested as an initiating factor for EpSCC. It has also been suggested that EpSCC may be more likely to develop in EcPV2 infected tissue because of raised levels of inflammation, which is associated with both tumorigenicity and papilloma virus infection^7,12^. However, it is difficult to separate cause from effect from these findings. The diagnostic and prognostic indicators rely on histopathological interpretation, whilst mechanisms of molecular carcinogenesis are not yet known.

We recently discovered that the activation of the Wnt pathway is an important feature of human penile squamous cell carcinoma^18^. The Wnt network is a highly evolutionarily conserved signalling pathway, known to play a role in cell homeostasis, differentiation, proliferation, development and motility. The Wnt pathway, directly and indirectly, also promotes gene transcription of numerous targets, many of which are transcription factors^19^. An intersection of the links between the Wnt pathway, cancer and inflammation is to be found in bowel diseases. Mutations in the Wnt pathway are predominant in human colon cancer^20^ and there is also emerging evidence that the Wnt signalling network is involved in the modulation of the inflammatory response, as reviewed recently^21^.

In this study, we investigated if aberrations in human penile cancer related proteins, thought, generally, although not exclusively, to be under the transcriptional control of the Wnt signalling pathway in horses. Because EcPV2 and inflammation have also been predicted as a risk factor in the development of EpSCC, we also wished to test for changes in protein expression in relation to the presence of inflammation and EcPV2 expression. To investigate this notion, we selected four proteins associated with Wnt signalling and human penile squamous cell carcinoma: Matrix Metalloproteinase 7 (MMP7), Cyclin D1, c-Myc, and Fos-like antigen 1 (FRA1). FRA1 is a member of the Fos family of proteins, which also includes Fos B, c-Fos, and Fra2^22^ and a target of transcription for the Wnt signalling pathway. The Fos proteins, along with proteins from the Jun family, make up the activator protein 1 (AP1) transcription factor^23^. There has been much interest in the role of aberrant expression of Fos proteins in multiple human cancers, including cancers of the liver, pancreas and ovaries^24–27^. We have previously shown the up-regulation of MMP7, Cyclin D1, c-Myc in human penile squamous cell carcinoma^18^.

In analysing cancer related proteins, putatively associated with the Wnt pathway, in EpSCC and their association with EcPV2 infection we may find dual benefit in creating an animal model of human disease as well as improving our understanding of an equine condition that causes significant morbidity. Using a cohort of EpSCC tissue, we conducted medium throughput protein staining and imaging and a quantitative approach previously established in our laboratory^18,28–31^ to investigate the hypothesis that viral-driven alterations in inflammation may play a role in EpSCC development.

## Results

### Pathology

**Figure 1:**
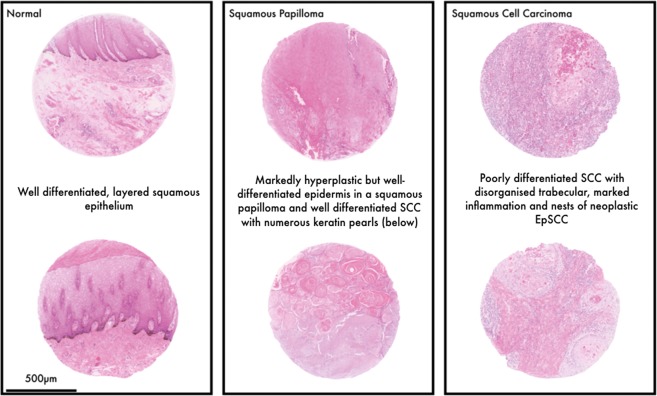
Histological grading of specimens. Representative, 1 mm diameter tissue core images of normal, squamous papilloma and squamous cell carcinoma. Tissue arrays were constructed containing tumour and tumour adjacent samples. The H&E section of this tissue array was used for classification of the disease by two expert veterinary pathologists. Pathological features are noted next to the tissue cores.

Histopathological evaluation of slides was used to define the cores as either Normal (NT), Hyp/Pap, or EpSCC (Fig. 1), the degree of differentiation (poorly-differentiated EpSCC (PDSCC), or well-differentiated EpSCC (WDSCC)) was also recorded for each tissue core. In addition, mitotic index (MI), a measure of cellular proliferation which describes the ratio of cells undergoing mitosis was also calculated (Table 1). In total, 13 out of 34 cases were well-differentiated tumours, whilst 20 were poorly-differentiated; MI was positively correlated with malignancy (p = 0.04). In well-differentiated tumours, mean MI was 9 ± 7 whilst in poorly-differentiated cases MI value was 23 ± 16 (mean ± SD).

**Table 1:** Mitotic index for tumour type and differentiation.

Tumour Type (n)	Mitotic Index		Significance
	Mean	Range	
EpSCC (34)	18	2–66	p = 0.04*
pP (11)	9	1–30	
Well-differentiated pSCC (14)	9	2–22	p < 0.001***
Poorly-differentiated pSCC (20)	23	3–66	

Number of samples (n) is recorded for each tumour and differentiation type. Mitotic index, defined as the total number of mitotic figures counted in ten x400 fields at 2.37mm^2^, was calculated by expert veterinary pathologists from H&E stained tissue array images. Mitotic index was positively correlated to malignancy (*p < 0.05).

### Quantification of inflammatory cells in EpSCC

**Figure 2:**
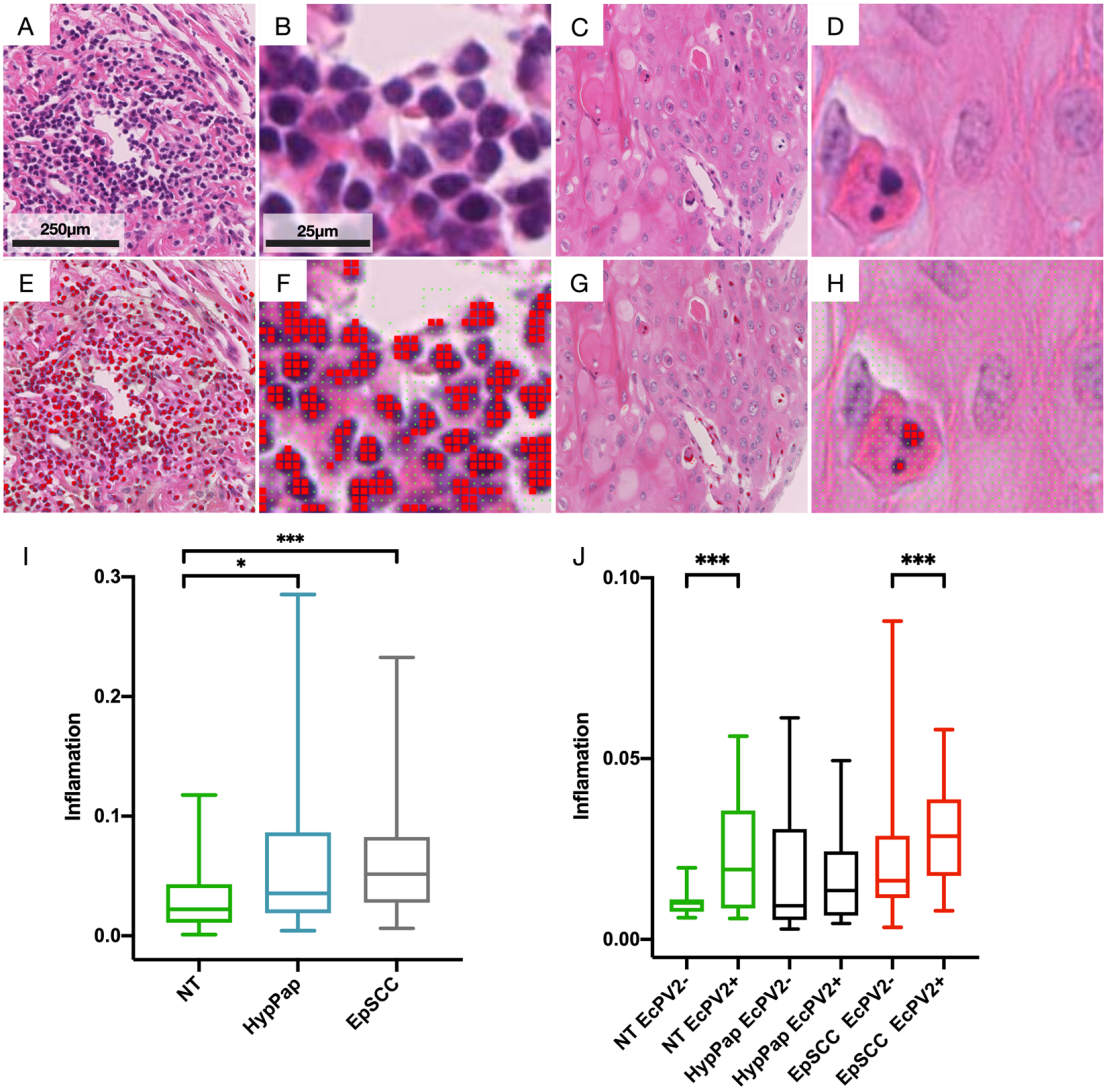
Quantification of inflammatory cells on H&E stained tissue cores. A CNN, consisting of two convolution layers and a fully connected layer, was trained on a dataset of 1200 images (11 × 11 pixels) of inflammatory cells and 1897 images of non-inflammatory areas of tissue. Predictions were made on a 11 × 11 pixel regions of tissue (**A**–**D**; H&E stained tissue core image, (**E–H**); H&E stained tissue core image with predictions overlaid). Green squares represent non-inflammatory predictions and the larger red squares represent the centroid of an image that has been predicted as inflammation (E-H). The final value for inflammation was calculated as a fraction of predicted inflammatory areas over total areas of tissue for each core (EpSCC EcPV2 + n = 23, NT EcPV2 + n = 14, EpSCC EcPV2− n = 60, NT EcPV2− n = 17). Box plots (I, J) were constructed in GraphPad Prism to compare levels of CNN calculated inflammation in different pathologies (I) and EcPV2 infection status (J) (Mann Whitney U, ***p < 0.0001, **p < 0.001, *p < 0.05).

Inflammation was estimated by using three independent methods: 1. A convolutional neural network (CNN) 2. Visual scoring by expert pathologists (SLP and ASB) and 3. Staining for cluster of differentiation 3 (CD3) protein followed by unbiased quantification (see methods). A CNN was trained on a training dataset of inflammatory cell images (Fig. 2 and Supplementary Fig. 1), the resulting classifier is likely to identify cells based on the intensity of the nuclear chromatin staining with haematoxylin and cell shape (roundedness). The trained CNN classifier was used to make predictions across each H&E stained tissue core on areas of pixels that contain an image of tissue, using a hue saturation value (HSV) threshold. The total number of positive predictions over total predictions was used as a measure of inflammation, per core (Fig. 2A–E; examples of H&E stained images, E – H; predictions from CNN analysis overlaid on the original, corresponding images). The cell types identified were predominantly lymphocytes and plasma cells, and the network rarely misclassified areas of tissue such as the dark regions (clumped chromatin) in the nuclei of non-inflammatory cells or keratinocyte nuclei. A significant difference in levels of CNN measured inflammation between NT and both Hyp/Pap (p < 0.05) and EpSCC (p < 0.001) (Fig. 2I) was also observed. A higher level of inflammation was also observed in tissue cores that were determined to be positive for EcPV2 (Fig. 2J; also see Supplementary Fig. 3 for a panel of H&E images). In EcPV2+ cases, levels of inflammation were significantly higher in both the NT and EpSCC cohorts (p < 0.001) (Fig. 2J).

Visual analysis and scoring by pathologists was also used to estimate inflammation and correlate to CNN analysis, described above. Each tissue core was assigned a score for staining (0 = no inflammation, absent (0%); 1= mild (>0% to <5%); 2 = moderate (≥5% to <40%) 3 = marked inflammation >40%) by the pathologists. It was noted in this analysis that inflammatory cells were most often located in the stroma/propria immediately adjacent to the tumour. This can be seen in the example images in Fig. 2A–F and Supplementary Fig. 2A-F. Furthermore, there was a significant increase in EpSCC versus NT inflammation (p < 0.05) and an increase in EcPV2+ versus EcPV2− inflammation score for both NT and EpSCC (p < 0.05).

CD3 staining was used as another measure of inflammation for correlation and comparison with CNN analysis. Serial sections of tissue arrays were stained using a DAB immunohistochemistry protocol with a CD3 antibody followed by imaging and automated quantification of DAB stain. The results showed that CD3 expression in EpSCC cores was significantly higher than NT cores (Supplementary Fig. 2G) and a significant increase was also observed in the inflammation in EcPV2+ cores for the NT and EpSCC samples compared to EcPV2− cores (Supplementary Fig. 2H).

The analysis by pathologists and the quantification of CD3 staining correlated well with observations obtained using the CNN method demonstrating an increased inflammation in EpSCC compared with NT (p < 0.05) and an increase in EcPV2+ versus EcPV2− inflammation score for both NT and EpSCC for all three methods of quantification (Supplementary Fig. 2G,H). This was further confirmed when concordance between the CNN (Supplementary Fig. 2I) and CD3 (Supplementary Fig. 2J) inflammation quantification versus pathologist scoring (CNN (bias; SD of bias: 0.95; 0.82), CD3 (bias; SD of bias: 0.64; 0.71)) using the Bland-Altman test.

### Protein expression in EpSCC

**Figure 3:**
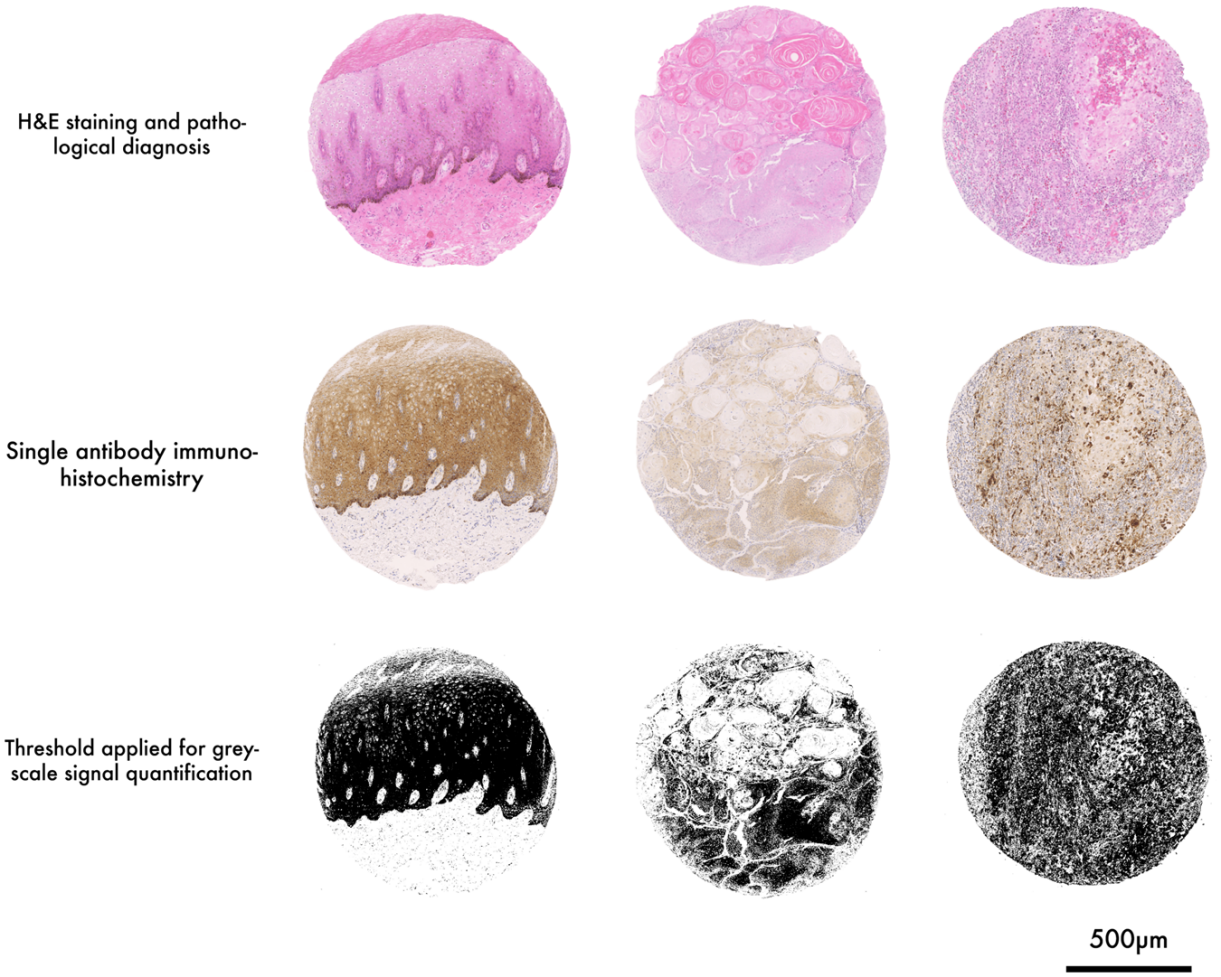
Tissue array cores, staining, imaging and analysis. Representative examples of H&E stain, single label DAB staining and grey scale converted images tissue cores are shown. Tissue arrays containing tumour and tumour adjacent EpSCC tissue were constructed using a Beechers manual tissue arrayer. Sister tissue array sections (4 µm) were stained with H&E (top row) or labelled (DAB) with either MMP7, FRA1, c-Myc, or Cyclin D1 (middle row) for quantitative protein analysis using a colour thresholding method in ImageJ software. The number of pixels within the thresholded image (binary mask, bottom row) is then counted and divided by the total amount of pixels that represent tissue in each 1 mm core. Analysis of tissue arrays, from which these examples are shown, were used for quantification and statistical comparisons are then made between the amount of FRA1 stain in tumour and tumour adjacent tissue using a Mann Whitney U test.

**Figure 4:**
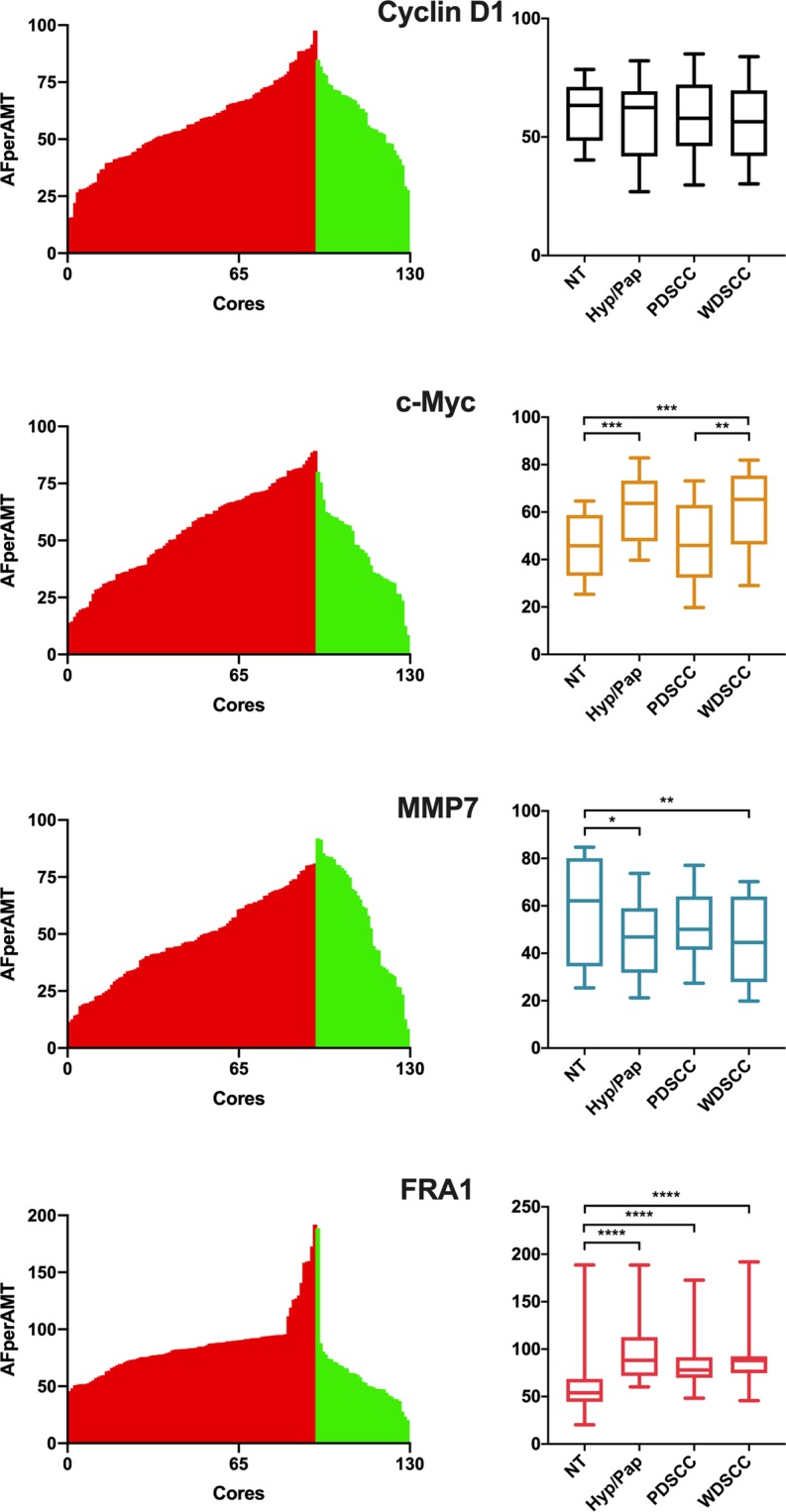
Protein expression in NT, Hyp/Pap PDSCC and WDSCC tissue cores. Tissue arrays were stained for MMP7, c-Myc, FRA1 and Cyclin D1. Tissue core images were thresholded for the amount of stain per amount of tissue for each tissue core (AFperAMT). Mountain plots (left) were constructed to show the protein expression for each tissue core with EpSCC tissue cores shown by red bars and normal tissue cores shown by green bars. A box plot of the AFperAMT in tumour and tumour adjacent cores was constructed to illustrate the change in expression in tumour tissue (right). Significance is represented by a * (Mann Whitney U, ****p < 0.0001, ***p < 0.001, **p < 0.005, *p < 0.05).

DAB label was detected for all four proteins in both NT and EpSCC equine tissue (Fig. 3, Supplementary Fig. 4). The protein expression of MMP7, c-Myc, and FRA1 was altered in WDSCC compared to NT (Fig. 4). In experimental controls, where tissue samples were incubated only with the secondary antibody and the primary antibody was omitted, no DAB signal was observed (see methods). MMP7 expression was significantly reduced in both Hyp/Pap (p < 0.05) and WDSCC (p < 0.01) tissue when compared to normal tissue. c-Myc expression showed an increase in both Hyp/Pap and WDSCC compared to NT (p = 0.0001). A significant difference in c-Myc expression was also observed between PDSCC and WDSCC (p < 0.005, Fig. 4). FRA1 protein expression showed an increase in all pathologies compared to NT (p < 0.0001).

**Figure 5:**
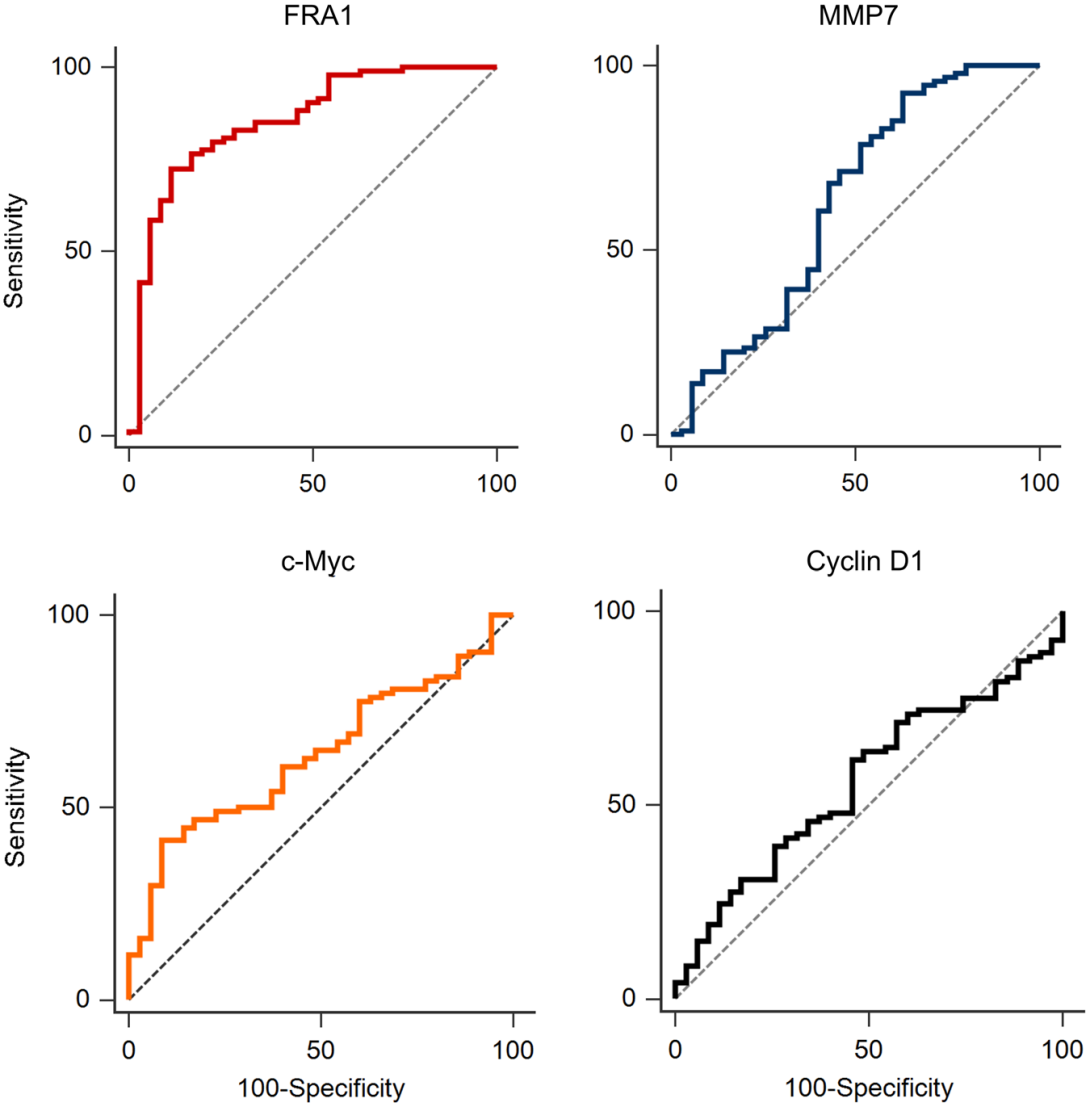
Receiver operating characteristics (ROC) curves. The AFperAMT for each tissue core was calculated using ImageJ software. AFperAMT for each core was then indexed according to the histopathological diagnosis provided independently by two veterinary pathologists evaluating a serial section of the TA stained with H&E. ROC curves were constructed using MedCalc software. Area under the curve (AUC) values and optimal sensitivity and specificity calculated using the Youden index (considered a measure of the best putative performance of a dichotomous diagnostic test) are as follows: FRA1; sensitivity = 72.34, specificity = 88.57, Area under the curve (AUC) = 0.854, criterion value > 74.1. c-Myc; sensitivity = 41.49, specificity = 91.43, AUC = 0.637, criterion value > 62.5. Cyclin D1; sensitivity = 61.70, specificity = 53.29, AUC = 0.559, criterion value > 61.5. MMP7; sensitivity = 92.56, specificity = 37.14, AUC = 0.627, criterion value <74.1.

ROC curves were constructed to test the sensitivity and specificity of protein expression as a predictor of EpSCC. The ROC curve for FRA1 revealed a moderate ability to correctly classify tumour or tumour adjacent tissue cores with an AUC of 0.85 (Youden index: sensitivity = 72.34, specificity = 88.57, criterion value > 74.10) (Fig. 5).

### EcPV2 viral nucleic acid specific PCR

Of the 43 formalin-fixed paraffin-embedded (FFPE) EpSCC tissue samples collected, 35 samples yielded DNA of acceptable quality for PCR using EcPV2 primers (Supplementary Table 1). Of these 35 samples tested, 13 showed a PCR product (224 bp) corresponding to the predicted size for the primers representing a section of the E1 region EcPV2 (Supplementary Fig. 5).

### Protein Expression in EcPV2 positive EpSCC

**Figure 6:**
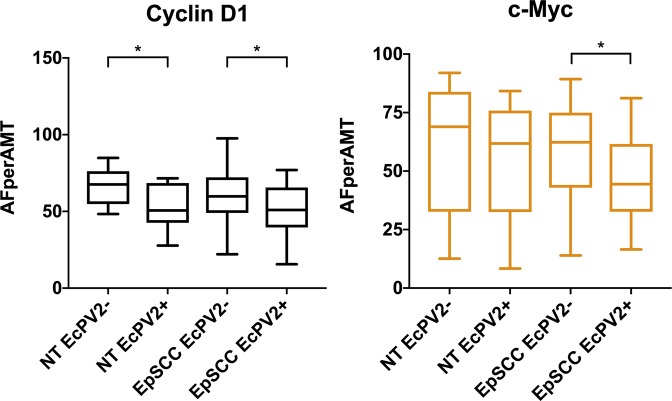
Protein expression in EpSCC and NT tissue cores identified EcPV2 positive or negative. Protein expression for Cyclin D1 and c-Myc was evaluated using a TA technique. Genomic DNA was isolated from EpSCC tissue samples and screened for the E1 region of the EcPV2 viral DNA. Tissue array cores were labelled according to their virus infection and expression levels were compared for tumour (EpSCC) and tumour adjacent (NT) tissue cores. Samples that were identified as either hyperplastic or papilloma (Hyp/Pap) were not included in this analysis. Tests of significance are labelled with a bracket (*p < 0.05). Other proteins that were tested, FRA1 and MMP7, showed no significant difference in expression correlated with virus presence.

From a sample set of 166 tissue cores we identified 47 samples that were EcPV2 + (EpSCC n = 23, NT n = 14) and 87 samples which were EcPV2− (EpSCC n = 60, NT n = 17; a panel of H&E images is also provided in Supplementary Fig. 3). Comparative analysis was conducted in EcPV2+ vs EcPV2−, between NT and EpSCC samples). There was a significant (p < 0.05) change in the expression of Cyclin D1 and c-Myc in tissue samples that were EcPV2 + (Fig. 6). The protein expression of Cyclin D1 was reduced in EcPV2 tissue samples in both the NT and EpSCC cohorts. The expression of c-Myc was reduced in EpSCC EcPV2+ samples only (Fig. 6).

### Quantitative colocalisation of two proteins

**Figure 7:**
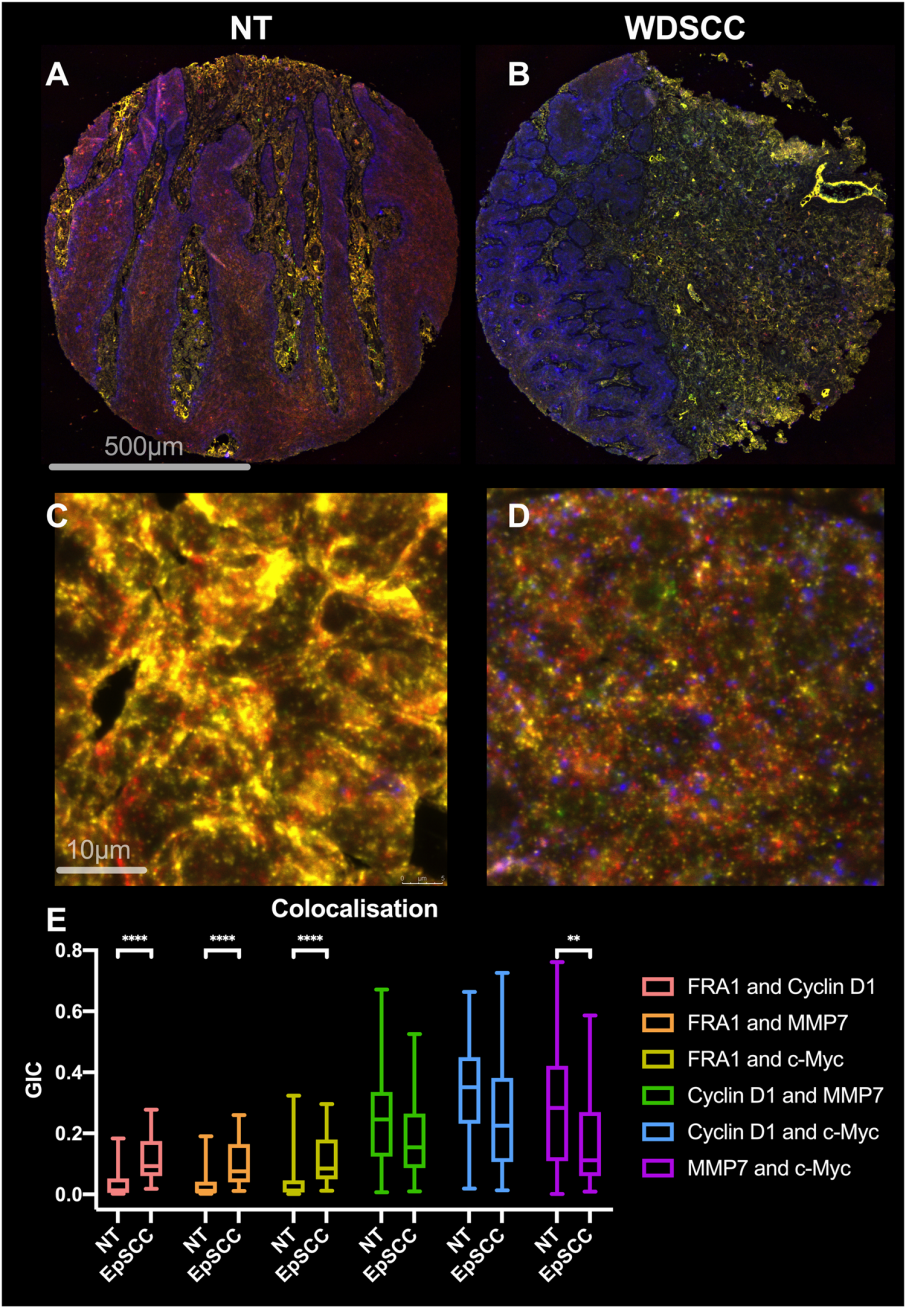
Colocalisation of FRA1, Cyclin D1, c-Myc, and MMP7. TA slides, labelled for Cyclin D1, MMP7, c-Myc, and FRA1 with FITC (488/517 nm, Blue), Cy3 (514/565 nm, Yellow), Cy5 (633/671 nm, Green), and Coumarin (405/470 nm, Red), respectively, were imaged at both low (10×, **A**,**B**) and high (40x with a 6x zoom, C and D) using a Leica TCS SP8 confocal microscope (n = 12, 6 EpSCC and 6 NT). High magnification, z stack, images (0.17 µm step size) were first deconvolved using Huygens software. The global intersection coefficient (GIC) was calculated between each channel as a measure of colocalisation of two proteins. Box plots and tests of significance were carried out using GraphPad Prism (Mann Whitney U, ****p < 0.0001, **p < 0.005).

Tissue array slides stained to detect multiple proteins were analysed using deconvolved, confocal images, to measure protein co-localisation by calculating the global intersection coefficient (GIC) in NT and diseased tissue cores (Fig. 7, Supplementary Figs. 6, 7). There was a significant change in GIC for FRA1/Cyclin D1, FRA1/MMP7, and FRA1/c-Myc (p < 0.0001) and MMP7/c-Myc (p < 0.005) in EpSCC tissue cores compared with NT cores.

## Discussion

We have previously found increased expression of the Wnt signalling targets Cyclin D1, MMP7 and c-Myc in human penile squamous cell carcinoma^18^. Here, we have analysed the expression of these proteins and FRA1, another known target of Wnt signal activation^32^, to further investigate this phenomenon in equine tissue. Although each biomarker was chosen for its known association with Wnt signalling with respect to four pathologies (NT, Hyp/Pap, PDSCC and WDSCC), the transcription of these genes is not exclusively under the control of Wnt/beta catenin transcription factor co-activator^33,34^. We showed that there were significant deviations from normal expression in c-Myc, MMP7, and FRA1 that may be further investigated as use as a biomarker of disease.

### Cyclin D1

Cyclin D1 belongs to the cyclin family and assists in regulation of cell migration and cell cycle progression by controlling G1/S phase transition and has a role in regulating cell proliferation and differentiation by binding to nucleolar receptors. Evidence implicates Cyclin D1 in the invasiveness and metastatic capability of urinary bladder tumours^35,36^.

### MMP7

MMP7 is part of the MMP family of peptidases, who have a major role in the breakdown of extra-cellular matrix in normal physiological processes, embryonic development, and reproduction and tissue remodelling. It is implicated in the aggressiveness and metastatic potential of bladder cancer^37–39^.

### c-Myc

c-Myc is a transcription factor that receives direct signals from Wnt as well as other pathways, being involved in cell proliferation, apoptosis and differentiation. It is implicated in bladder cancer, being overexpressed and seen as a potential target for chemotherapeutics^40–42^.

### FRA1

FRA1 plays important roles in various biological processes, including inflammation, transformation, proliferation and metastasis^43^. It is a direct target of the Wnt signalling pathway^44^ and the Interleukin (IL) signalling pathway *IL-*17RA^45^. FRA1 is implicated in the development and upregulation of various human malignancies including lung, brain, breast and bladder^43,46–48^.

Additionally, FRA1 is implicated in human research to regulate inflammatory processes, leading to disease such as Rheumatoid and osteoarthritis^49^. Considering the emerging importance of FRA1 in cancers, we asked the question, is FRA1 expression altered in EpSCC tissue?

FRA1 is a downstream target of the Wnt/beta-catenin signalling pathway, which is implicated in the development of several malignancies^43^. We discovered that a number of Wnt targets of transcriptions were upregulated in human penile squamous cell carcinomas, suggesting a role for this pathway in penile carcinoma^18^. Wnt as a core pathogenetic driver of squamous cell carcinoma in humans has also been observed in other tissues^50,51^. Additionally, aberrant expression of FRA1 through Wnt activation has also been shown in glioma cells, indicating a linkage^43^.

Previous studies have indicated that EcPV2, a non-enveloped DNA virus, is associated with the presence of EpSCC. Here, we have directly corroborated this observation by screening 36 EpSCC samples for the presence of the viral infection, finding its presence in 13 cases.

Pathological observations showing inflammation accompanying a significant number of EpSCC samples is in keeping with other studies^7,12^, an observation that is also seen in human penile squamous cell carcinoma^52^. This may support a model of inflammation as a possible aetiological factor for EpSCC, with infection precipitating the inciting inflammatory processes. If this is the case, EcPV2 is an indirect precipitant of EpSCC and therefore the increased expression of FRA1 could be explained by its role in the immune response. FRA1 overexpression in transgenic mice models has been shown to have increased inflammatory cell infiltration in the liver^49^.

### Inflammation, EcPV2 and EpSCC

The association between chronic inflammation and oncogenesis has been a subject of intensive discussion and research for some time^53^. Dvorak^54^ suggested that tumours are wounds that don’t heal. The relationship between papilloma virus infection and inflammation is also well documented^55^. There is evidence, both in human and horse, associating papillomavirus infection with squamous cell carcinomas of the penis^7,15,56^. The relationship between infection and dysplasia (a pre-malignant change) is well-characterised in the case of human cervical cancer, where HPV is found in over 99% of cervical cancers^13^. Integration of viral DNA into local tissue disrupts cellular processes that may lead to malignancy. Similar variations in HPV association are seen in equine cancers where rates vary between 45 and 100%^7,15^ suggesting that there are other potential causes of mucosal squamous cell carcinomas in the horse, with UV-radiation, smegma accumulation and breed factors have been suggested^4,57^. We have used a CNN method of inflammatory quantification as a fast and cost-effective method of comparing inflammatory cell levels in H&E images, complimenting immunohistochemical and scoring methods employed by pathologists. The comparative methods show a good level of concordance (Supplementary Fig. 2).

Cluster of differentiation 3 (CD3) is a protein complex composed of epsilon, gamma, delta and zeta polypeptide chains. The CD3 complex binds to T cell receptors (TCR) and is considered a T cell marker^58^. It is known that CD3 expression is associated with inflammation. For example, the number of CD3 + cells was shown to be increased in patients with rheumatoid arthritis^59^. CD3 has thus been used as a marker for inflammation in tissues for many years^60^. CD3 expression in equine tissue was first described by Blanchard-Channell^61^ in mononuclear leucocytes and lymphoid tissue. Both the scoring method used by pathologists and CD3 quantification support the idea that the CNN methodology described here may be used to detect inflammation levels between tissue samples.

Utilising the CNN methodology, we found a positive correlation between inflammation and EcPV2 and EpSCC. There was an increase in inflammation in both NT EcPV2+ and EpSCC EcPV2+ samples compared with EcPV2− samples of the same cohort. We can perhaps, speculate that immune response to EcPV2 is a factor contributing to EpSCC progression. The CNN approach could be applied to other human (e.g. prostate^28,30^) and animal cancers^29^.

## Materials and Methods

### Sample selection and tissue array construction

Only FFPE tissue from pathology archives was used in this research. The tissue samples were collected for diagnostic and/or treatment purposes. Archival tissue collection for use in research were subject to institutional ethical review. Ethical approval for the use of equine tissue was granted by the Clinical Research Ethical Review Board (CRERB) of the Royal Veterinary College, University of London. All methods were performed in accordance with the relevant guidelines and regulations. Forty-three FFPE tissue samples of histologically diagnosed EpSCC were obtained from the pathology archives of the Royal Veterinary College and University of Bristol, UK. Samples originated as either incisional or excisional biopsies taken for diagnostic purposes for the animal (mean age 19, range 8–33 years) (Supplementary Table 1). Clinical data, where present (histological diagnosis, treatment and outcome) were collated with sample labels coded to maintain the blind experimental design. Samples were sectioned, stained with haematoxylin and eosin (H&E), and independently classified by two veterinary pathologists (SLP and ASB) for both disease process and differentiation, defined here as well-differentiated and poorly-differentiated. Areas of EpSCC (n = 34), tumour-adjacent control (n = 23), and Penile Papilloma (pP) (n = 11) were marked on the tissue block for tissue array construction.

A sample size calculation was made incorporating parameters from previously described studies^18,28–30^; the minimum tumour and control group sizes were 21 and 7 respectively to ensure adequate statistical power to detect differential expression of protein signal. Tissue arrays were constructed using a Beecher Instruments Microarrayer (MTA-1 tissue arrayer, Beechers Instruments, Sun Prairie, WI, USA). Tissue samples were cored from the areas marked by the pathologists. Tissue arrays (TA) were constructed with paired control (defined here as tumour-adjacent) and tumour samples on the same array (where applicable). Tumour tissue samples were repeated in triplicate and tumour adjacent specimens in duplicate. A total of five TA paraffin blocks were constructed with 1 mm cores at 3 mm intervals. Random core positioning ensured replicated samples were distributed evenly across the array to control for staining procedures. A (4 µm) section from each TA was stained with H&E and again each core was histopathologically examined and assigned a classification of either: NT, Hyp/Pap, PDSCC, or WDSCC.

### Immunohistochemistry

The antibodies, Cyclin D1 (sc-718 Santa Cruz), MMP7 (ab4044 Abcam), c-Myc (NCL-cmyc Novacastra/Leica), and FRA1 (ab117951 Abcam) were optimised for pH dependence, antigen retrieval, and concentration. Following optimisation, single label, 3,3-diaminobenzidine-horse radish peroxidase (DAB) staining was performed for each protein (Supplementary Fig. 4) at the following dilutions 1:500, 1:250, 1:250, 1:1000 respectively.

All tissue array slides were stained with the Bond Max automated staining system (Leica Biosystems, Milton Keynes, UK) in a single experiment to reduce inter-slide variations in staining. Control staining was carried out where no primary antibody was used (Supplementary Fig. 8). A multi-label stain was performed with Cyclin D1, MMP7, c-Myc, and FRA1 which, after binding to the respective Horse Radish Peroxidase secondary antibody, were visualised following the principle of Toth and Mazey using the following fluorescent tyramides; FITC (488/517 nm), Cy3 (514/565 nm), Cy5 (633/671 nm), and Coumarin (405/470 nm), respectively.

### DAB stain Imaging and analysis

Single label DAB stained slides were imaged at 40x magnification using a Nanozoomer slide scanner (Hammamatsu Photonics UK Ltd, Welwyn Garden City, UK) and an image of each individual tissue core was exported and indexed according to its configuration on the TA (Supplementary Fig. 4). Images were analysed using ImageJ software^62^ and algorithms developed in our laboratory^18,28^. Areas containing the DAB stain (brown) were segmented on hue, saturation, and brightness. Areas containing tissue were identified on a duplicate image by conversion to 8-bit and application of a threshold to only measure pixels that are not the same intensity as the background. Thresholds were optimised on a training set of images (n = 20 for each antibody) before being applied to the whole image set for standardization of quantitation. A macro was compiled for unbiased quantitation to measure both the amount of DAB signal and the amount of tissue. Areas of folded tissue were excluded from analysis. Results are expressed as the amount of signal divided by the amount (AMT) of tissue and fitted to a probit regression (Area Fraction, AFperAMT). Mountain plots and Box plots illustrating changes in protein expression were created in GraphPad Prism. Tests of statistical significance between tumour and tumour adjacent tissue cores were conducted using a Mann Whitney U test (Medcalc software). A receiver operating curve (ROC) curve was constructed to test the ability of the quantified protein labels to correctly classify between tumour and tumour adjacent tissue cores.

### EcPV2 PCR and Amplicon Sequencing

Genomic DNA was isolated from FFPE equine penile tissue using a QIAamp DNA FFPE Tissue Kit (Qiagen, Manchester, UK). Primers for the E1 region EcPV2 (Genbank sequence EU503122.1) were used which have previously been used to show the presence of the viral DNA in equine penile tissue (EcPV2 Forward: ATTACCGCAGAGCGGAGATG, EcPV2 Reverse: GCTGGACTTGCCAGTGTTTG)^7^. PCR reactions containing Taq PCR Master Mix (Qiagen, Cat no: 201445) 20 µL, 0.1 µM EcPV2 forward and reverse primers, and 5 µL template were amplified at 94 °C for 3 minutes and 30 cycles of 94 °C for 1 minute, 57 °C for 1 minute, and 72 °C for 1 minute, followed by 72 °C for 5 minutes. DNA extraction from amplicons was carried out with a QIAquick Gel Extraction Kit (Qiagen, Manchester, UK). Amplicon sequencing was undertaken by Geneservice Source BioScience PLC (Nottingham, United Kingdom).

### Quantification of inflammatory cells in EpSCC

To test for any association of tumorigenicity and EcPV2 infection with inflammation, areas within the tissue cores with inflammatory cell infiltration were assessed (SP and ASB). These areas were used to train a convolutional neural network (CNN); the CNN was implemented using Keras^63^ and Tensorflow^64^ with an architecture consisting of two convolution layers, a fully connected layer and an output layer. The resulting CNN was trained on a NVidia 2080ti GPU. Training datasets were constructed using 15% of the total number of H&E tissue cores (n = 149). 11 × 11 pixel images of inflammatory cells (n = 1200) and non-inflammatory areas of tissue (n = 1897) were manually curated and used for CNN training. The non-inflammatory areas category also included areas of out of focus and fragmented tissue that are likely to cause the CNN to misclassify an input image^65^. Predictions were then made on the entire dataset by first pre-processing whole core images to identify areas of tissue, using a colour threshold, and then iteratively making predictions on 11 × 11 pixel regions of tissue. The final value of inflammation was calculated as a fraction of inflammatory areas over non-inflammatory areas for the entire tissue core. Two methods were used to validate the CNN method of quantification. Firstly, H&E-stained images of each core were examined by two board-certified veterinary pathologists (SLP and ASB) and a consensus of total histological inflammatory score was assigned for each tissue based on the percentage of inflamed surface area in the core^66^ as follows: 0 = absent (0%); 1 = mild (>0% to <5%); 2 = moderate (≥5% to <40%); 3 = marked (≥40%). All inflammatory cells (lymphocytes, plasma cells and neutrophils) were assessed equally. Secondly, all tissue arrays were stained for CD3 (Leica Biosystems: CD3–565-L-CE), a T cell marker^67^ using quantitative immunohistochemistry (see above). DAB signal for CD3 staining was quantified independently for each tissue core using a quantitative measure to determine the fraction of stained tissue. Comparisons were made between the CNN method, analysis by pathologists, and levels of CD3 staining (expressed as mean ± SD) and Mann Whitney U test comparisons of significance. Bland-Altman tests were also used to assess concordance between CNN analysis and comparison with scoring by pathologists and CD3 expression using quantitative immunohistochemistry. To do this comparison, CD3 and CNN quantification data was first scaled using the difference of the mean value, to fit in the pathologist score range. Bland-Altman was calculated using GraphPad Prism and plotted as (A –B) versus the mean of A and B, where method A is the scoring of inflammation by pathologist and method B is CD3 or CNN inflammation quantification, respectively.

### Quantitative colocalisation

Multi-labelled tissue slides were imaged using a Leica SP8 confocal microscope with a 40 × 1.4 NA objective and a 6x zoom was applied during image acquisition (equivalent to 4 times over sampling to satisfy Nyquist criteria for deconvolution). Five Z-stack images with a z-step size of 0.17 µm (~ 20–28 z-sections per sample area) were taken from each tissue core (n = 12, 6 EpSCC and 6 NT). Images were deconvolved using Huygens software (Scientific Volume Imaging) and the global intersection coefficient (GIC) was calculated for each pair of channels in the 4-channel image. Representative images are shown in Supplementary Figs 6,7. The Mann Whitney U test was used for significance tests between EpSCC and NT tissue cores.

## Supplementary information

Supplementary Information.

## Data Availability

The datasets generated and analysed during the current study are available from the corresponding author on reasonable request.
