# Supplementary material for: Equine penile squamous cell carcinoma: expression of biomarker proteins and EcPV2
Source: Sci Rep. 2020 May 12;10:7863. doi: 10.1038/s41598-020-64014-3 (PMC7217868; doi:10.1038/s41598-020-64014-3)
Supplement: Supplementary file 1 — Supplementary Information. [file 41598_2020_64014_MOESM1_ESM.pdf]

Supplementary Information

Equine penile squamous cell carcinoma: expression of biomarker proteins and EcPV2

Callum Arthurs[1], Alejandro Suarez-Bonnet[2], Claire Willis[2], Boyu Xie[1], Natalie Machulla[1], Tim S. Mair[3], Kevin Cao[1], Michael Millar [4], Christopher Thrasivoulou[5], Simon L. Priestnall[2] & Aamir Ahmed[1]*

[1] Prostate Cancer Research Centre at the Centre for Stem Cells and Regenerative Medicine, King's College London, London, United Kingdom

[2] Department of Pathobiology and Population Sciences, Royal Veterinary College, Hertfordshire, UK

[3] Bell Equine Veterinary Clinic, Maidstone, UK

[4] Queen's Medical Research Institute, University of Edinburgh, Edinburgh, United Kingdom

[5] Research Department of Cell and Developmental Biology, The Centre for Cell and Molecular Dynamics, Rockefeller Building, University College London, London, United Kingdom

* aamir.ahmed@kcl.ac.uk (AA)

Year Diagnosed	Age	Breed	EcPV2
2008		New Forest Pony	-
2008	12	Pony	-
2008	22	Pony	+
2008	15	Appalosa	+
2009	22	Shetland	-
2009	26	Cob	-
2009	19	Cob	-
2009	11	Welsh D	-
2009	19		-
2010	16	Cob	+
2010	17	Pony	N/A
2010	30	New Forest X	N/A
2010	16	Cob	N/A
2011	19	Welsh D	-
2011			-
2011	23	Pony	-
2011			-
2011		New Forest X	-
2012	22	Irish Sports Horse	-
2012			+
2012		Irish Draught	-
2012			+
2012			+
2012	14	New Forest X	-
2012	18	Connemara X	N/A
2013			+
2013	8		+
2013		Pony	-
2013	25		-
2013	33	New Forest X	N/A
2013	19	Connemara X	N/A
2013	8	Irish Draught	N/A
2014		Thoroughbred X	-
2014			-
2014			-
2014			+
2014	23	Shetland	+
2014		Cob	+
2014		Irish Sports Horse	+
2014		Pony	-
2014	21	Pony	N/A
2015		Warmblood	-
2015			+

Supplementary Table 1. Sample information

Samples of surgically excised EpSCC tissue were collected from the Royal Veterinary College between 2008 and 2015. Age and breed of the sample where available was collated on collection of the sample. EcPV2 presence was determined by PCR amplification of a section of the E1 region of EcPV2. Samples listed as N/A did not yield sufficient DNA quality for the EcPV2 screen.

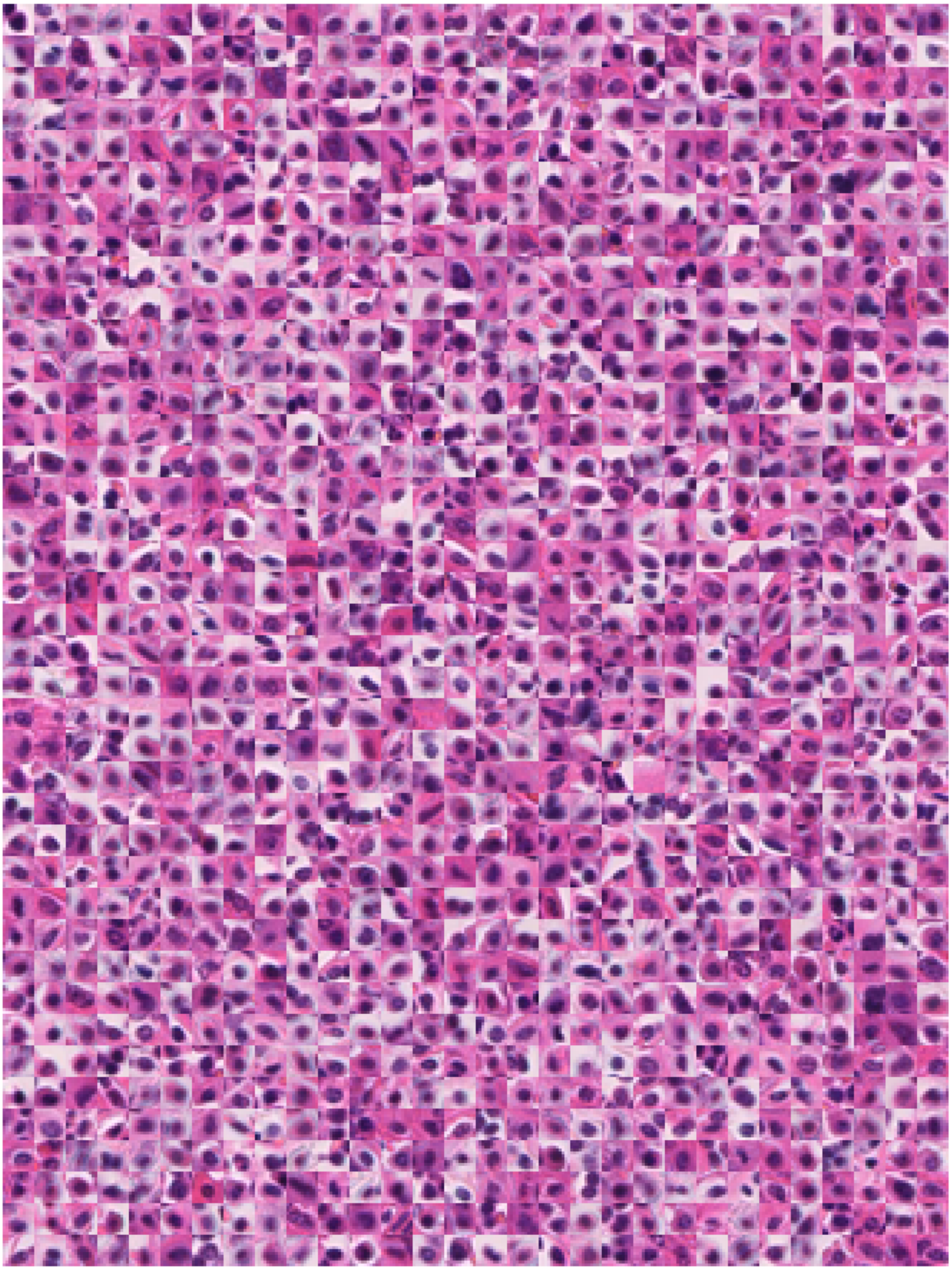

Supplementary Figure 1. Inflammatory cells used for CNN training.

Inflammatory cells (n=1200), comprising predominantly lymphocytes and plasma cells, were manually selected from 15 tissue core images, stained with H&E and imaged using a Hammamatsu Nanozoomer bright-field slide scanner at 40x magnification. 22x22 pixel images of each inflammatory cell were saved to a numpy array for CNN training.

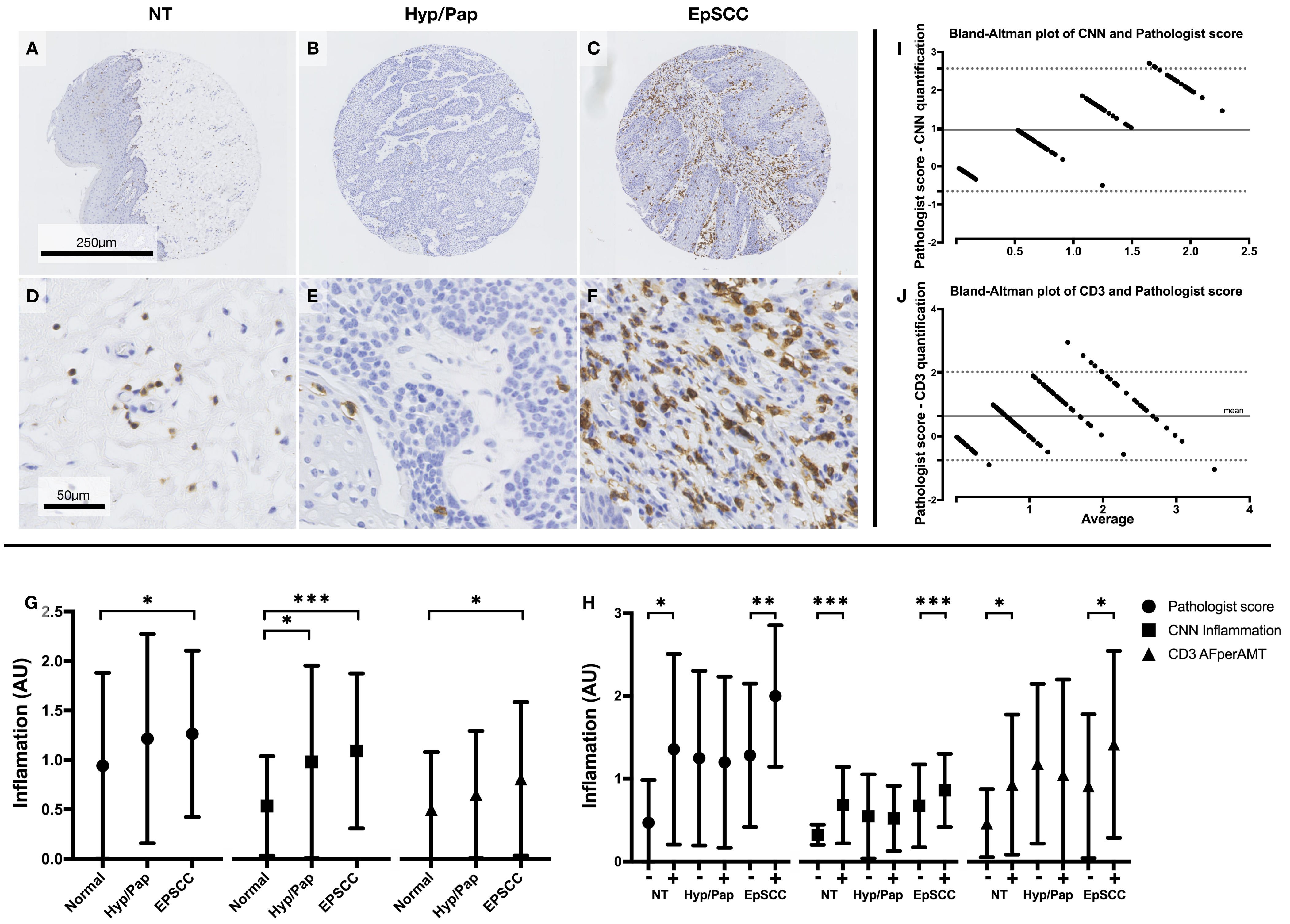

NT
Hyp/Pap
EpSCC
A
B
C
250µm
D
E
F
50µm
I
Bland-Altman plot of CNN and Pathologist score
Pathologist score - CNN quantification
J
Bland-Altman plot of CD3 and Pathologist score
Pathologist score - CD3 quantification
mean
Average
G
Inflamation (AU)
Normal
Hyp/Pap
EPSCC
H
Inflamation (AU)
NT
Hyp/Pap
EpSCC
Pathologist score
CNN Inflammation
CD3 AFperAMT

Supplementary Figure 2. Comparison between CNN and CD3 quantification.

A serial section (4µm) of the tissue array was labelled immunohistochemically for CD3 (Leica Biosystems: CD3-565-L-CE), a T cell marker. A – F, Slides were imaged at 40X magnification on a Hamamatsu slide scanner. CD3 DAB signal was quantified using a semi-automated method for all cores (EpSCC EcPV2+ n=23, NT EcPV2+ n=14, EpSCC EcPV2- n=60, NT EcPV2- n=17). Pathologist scoring was carried out via visual examination of each core by two board-certified veterinary pathologists (SLP and ASB) and a consensus total histological inflammatory score was reached. Scores were allocated as follows: 0; absent (0%), 1; mild (> 0% to < 5%), 2; moderate (≥ 5% to < 40%) and 3; marked (≥ 40%). Plots of mean and standard deviation were constructed using GraphPad Prism to compare results across CNN quantification, CD3 quantification and pathologist scoring of inflammation. CNN and CD3 values were normalised for presentation. G, comparison of inflammation levels between NT, Hyp/Pap and EpSCC cores. H, comparison of inflammation between samples containing EcPV2 positive and negative status. In every experiment a significant change in inflammation was observed between; NT and EpSCC, NT EcPV2- and NT EcPV2+, EpSCC EcPV2- and EpSCC EcPV2+ (Mann Whitney U, *** = p<0.0001, ** = p<0.005, * = p<0.05). Bland-Altman plots were constructed to measure concordance of the CNN (I) and CD3 (J) measurements of inflammation versus the pathologist scoring (CNN (bias; SD of bias: 0.95; 0.82), CD3 (bias; SD of bias: 0.64; 0.71). Dotted line represents 95% confidence interval. Plots were made using GraphPad Prism. For Bland-Altman test the data was transformed by scaling to allow comparisons between CNN, scoring by pathologists and CD3 expression analysis.

A

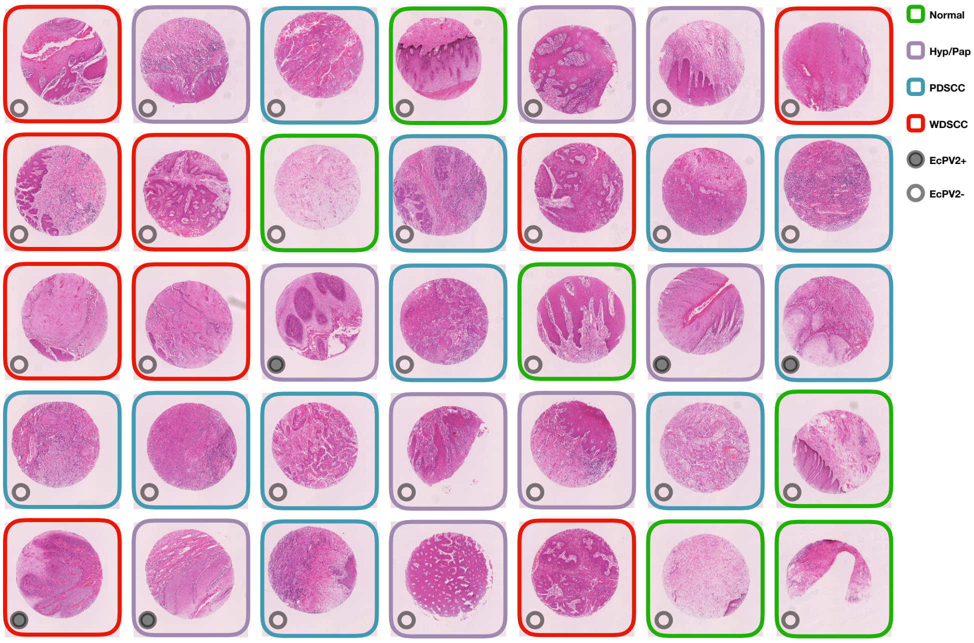
Normal
Hyp/Pap
PDSCC
WDSCC
EcPV2+
EcPV2-

B

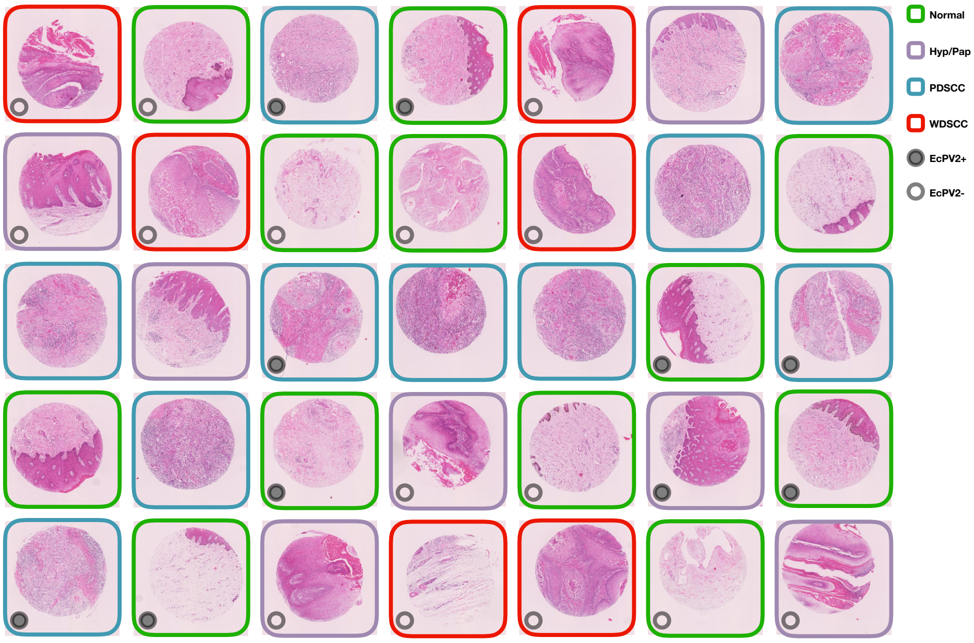
Normal
Hyp/Pap
PDSCC
WDSCC
EcPV2+
EcPV2-

C

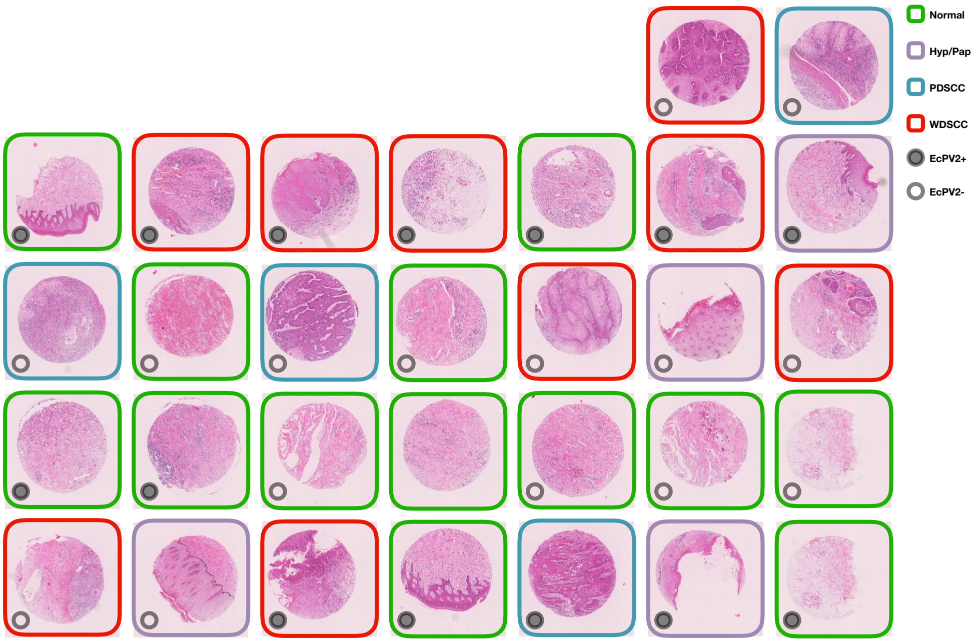
Normal
Hyp/Pap
PDSCC
WDSCC
EcPV2+
EcPV2-

D

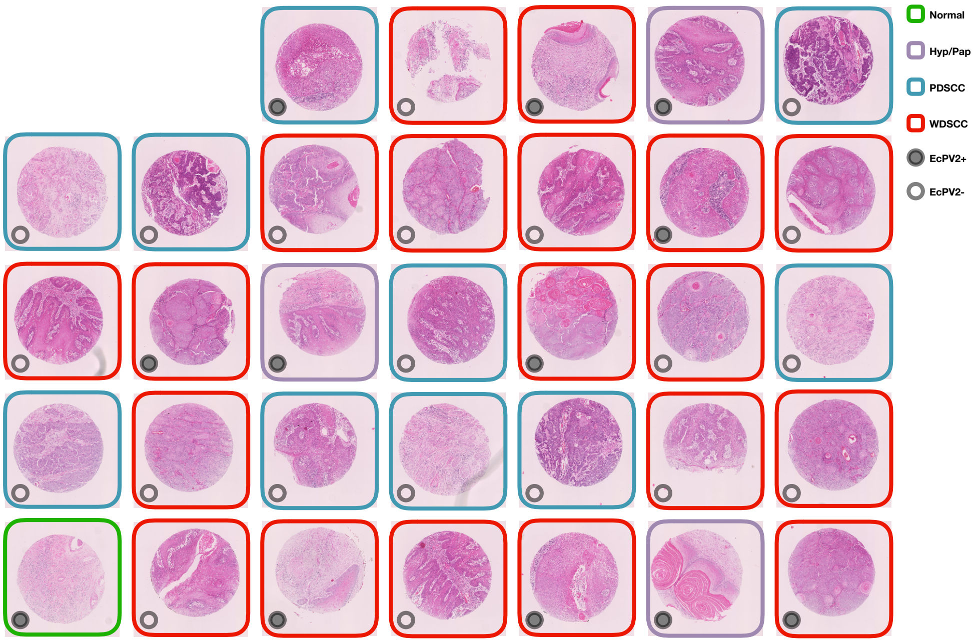
Normal
Hyp/Pap
PDSCC
WDSCC
EcPV2+
EcPV2-

E

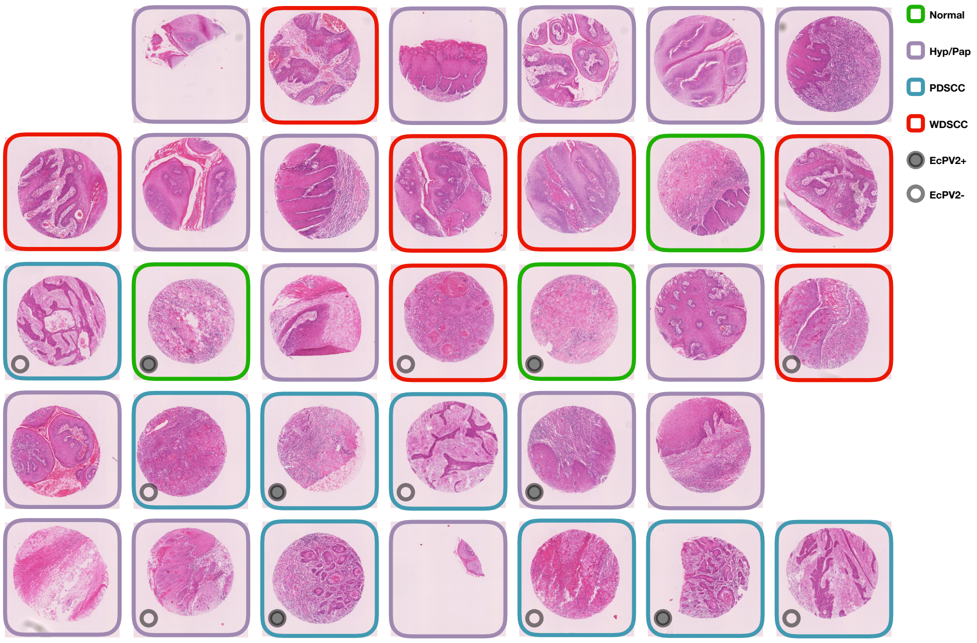

Supplementary Figure 3. H&E tissue array image with tissue cores labelled for Normal (green borders), Hyp/Pap (purple borders), PDSCC (blue borders), and EpSCC (red borders). EcPV2 disease was screened for using PCR. Shaded circles represent EcPV2+ tissue cores. Where there is no circle the DNA quality was not sufficient to carry out the PCR reliably.

MMP7

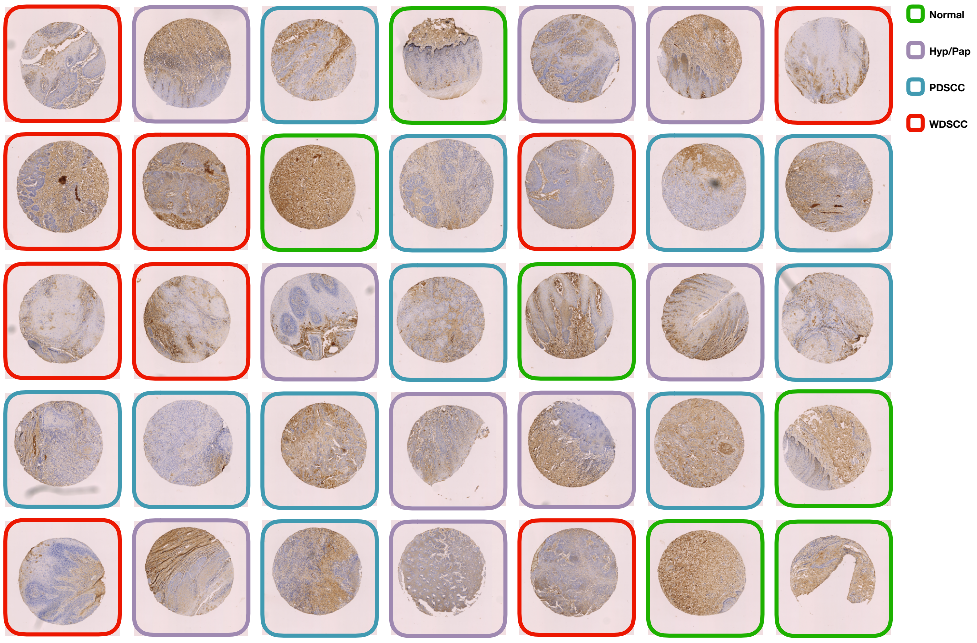

FRA1

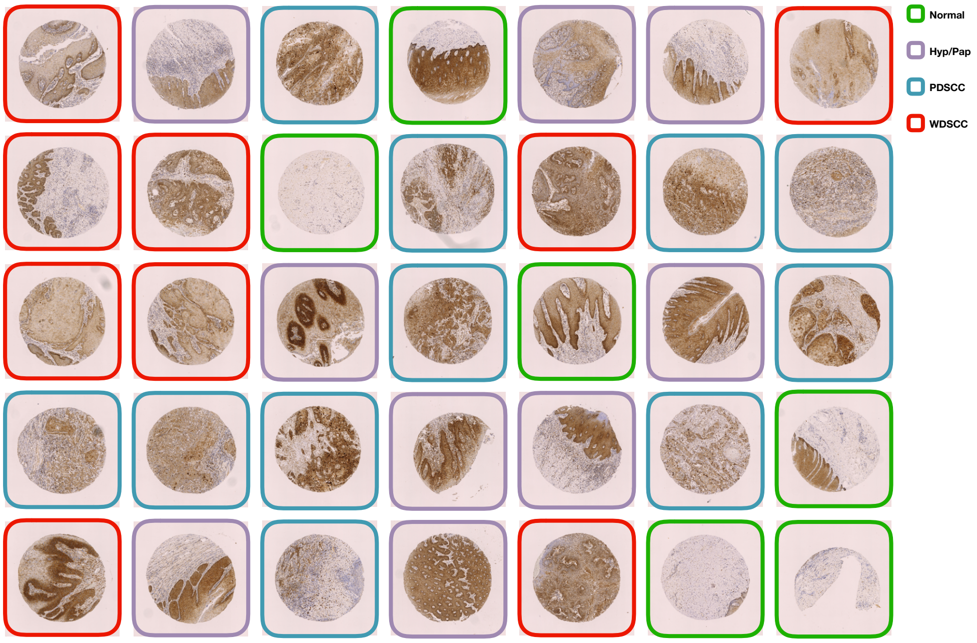

c-Myc

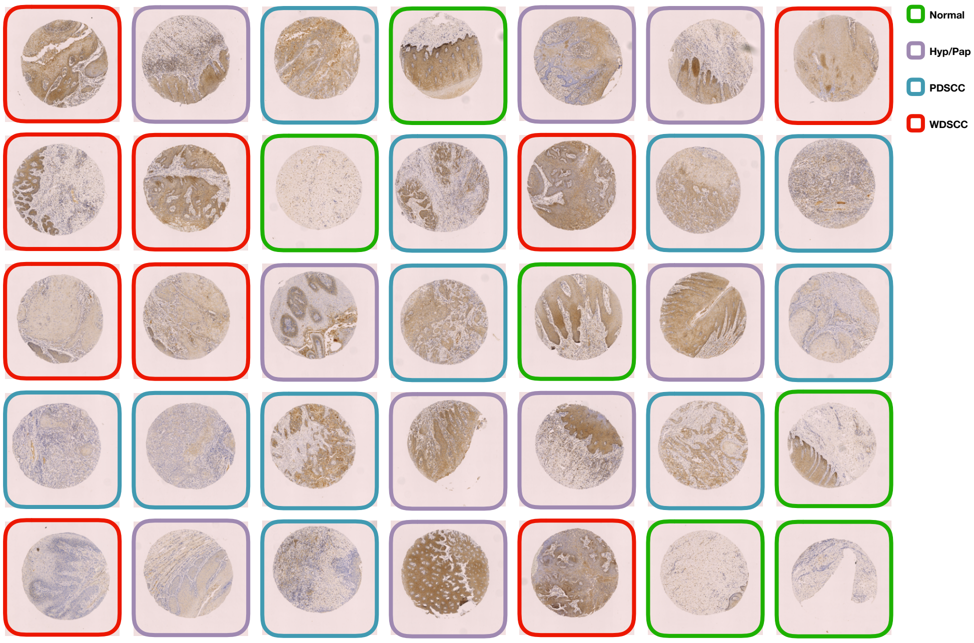

Cyclin D1

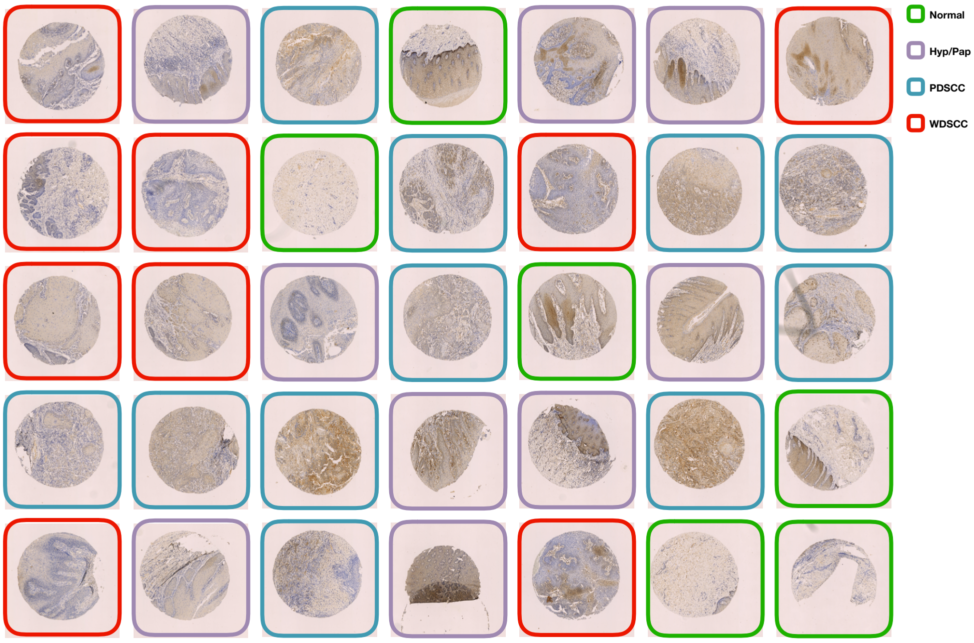

Supplementary Figure 4. Tissue array image with tissue cores labelled for Normal (green borders), Hyp/Pap (purple borders), PDSCC (blue borders), and EpSCC (red borders). Tissue arrays were constructed using tissue collected from excised EpSCC tissue and checked for the presence of tumour by two expert veterinary pathologists.

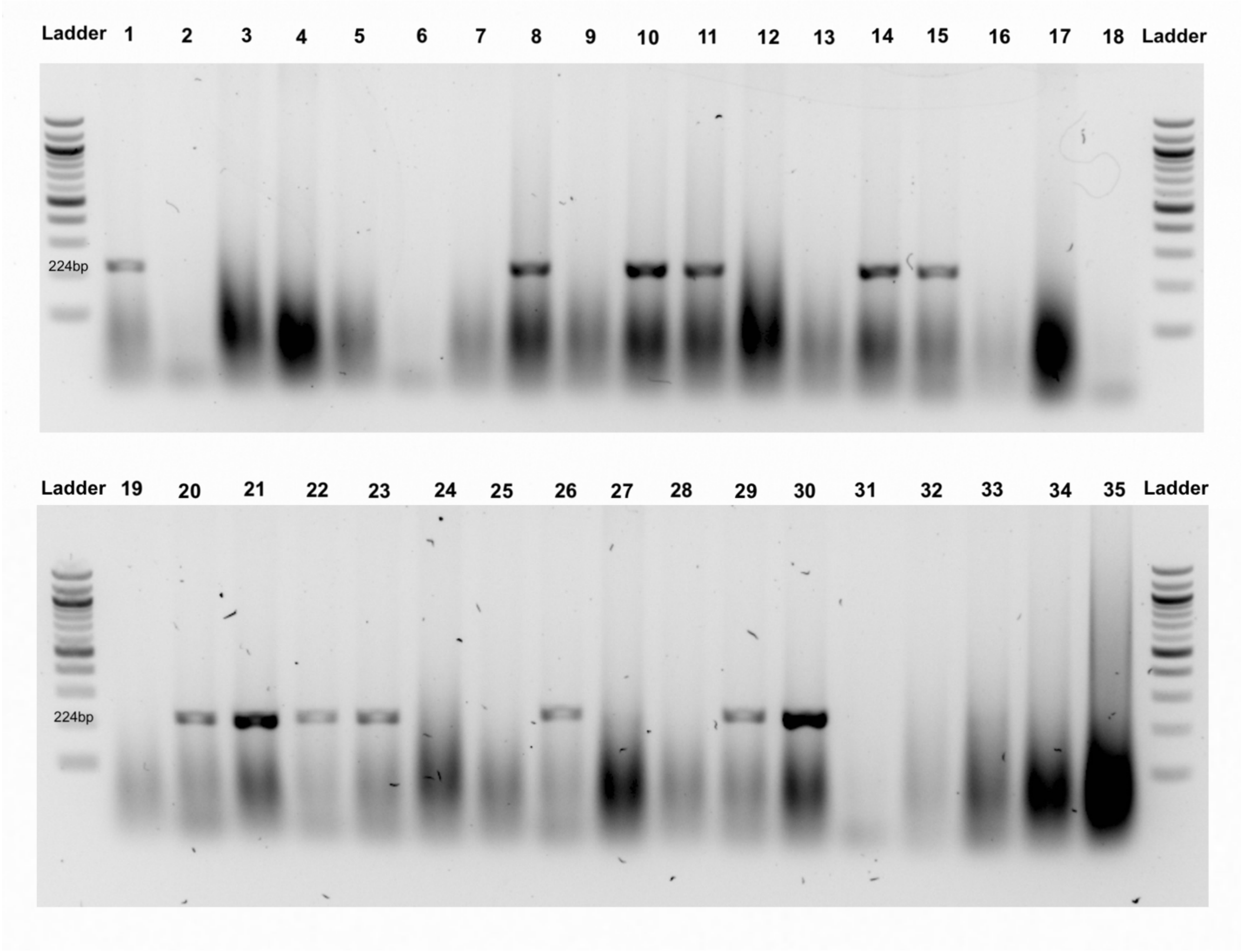

Supplementary Figure 5. Image of DNA gel.

Genomic DNA was isolated from FFPE tissue samples and a PCR reaction was carried out with primers to amplify a subset of the E1 region of the EcPV2 viral DNA. Samples were run on a 2% agarose gel alongside a 100bp ladder. The presence of a band at 224bp is representative of the EcPV2 infection. Sample numbers correlate to the sample index in Supplementary table 1.

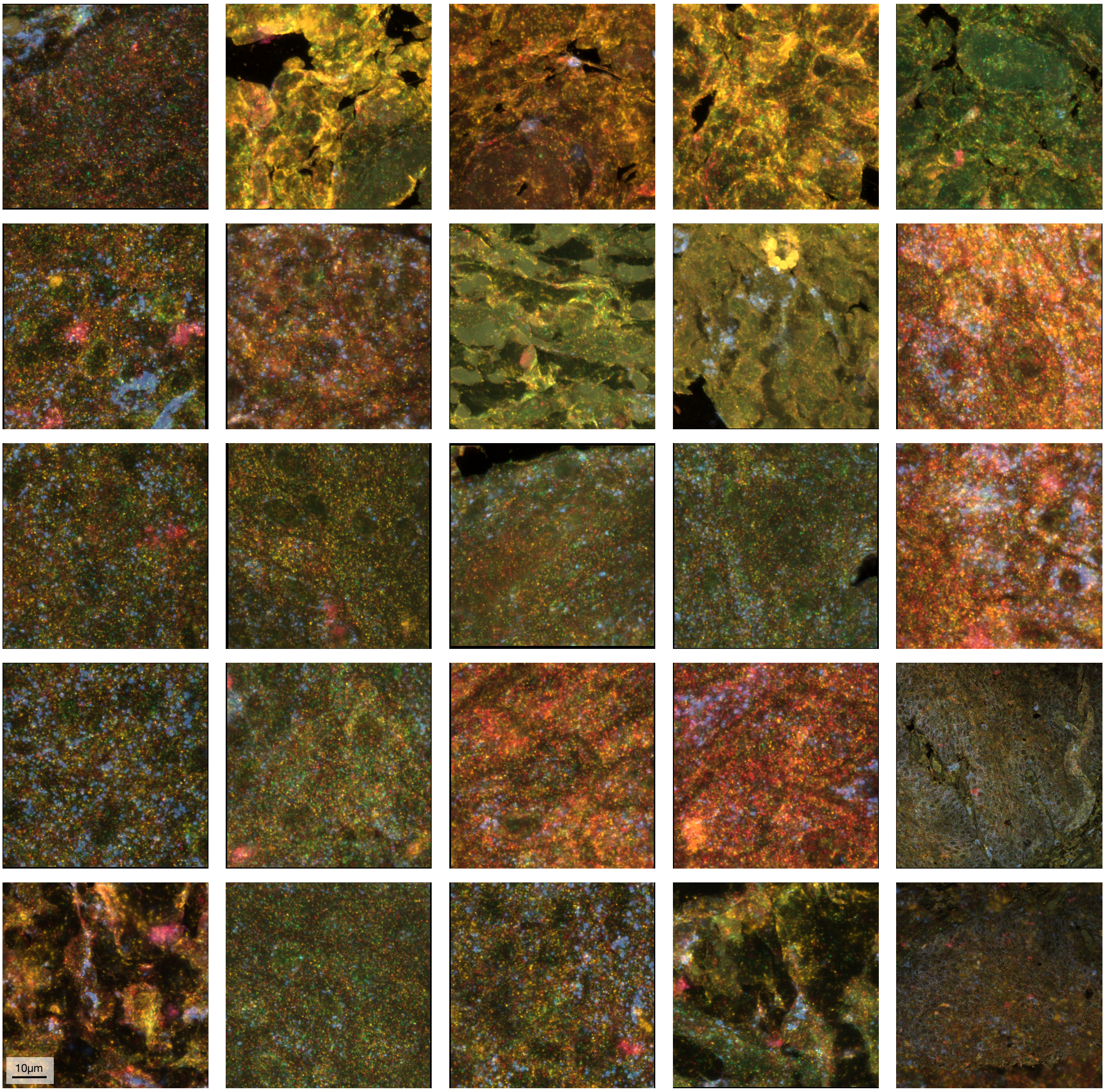

Supplementary Figure 6. Representative colocalisation images

Tissue array slides were multi-labelled for FITC (488/517 nm, Blue), Cy3 (514/565 nm, Yellow), Cy5 (633/671 nm, Green), and Coumarin (405/470 nm, Red) for Cyclin D1, MMP7, c-Myc, and FRA1 respectively. Regions of each tissue core were chosen at random (n=12, 6 EpSCC and 6 NT). High magnification images (40X oil objective with 6X digital zoom) were taken using a Leica TCS SP8 confocal microscope. Deconvolution was carried out in Huygens Professional software.

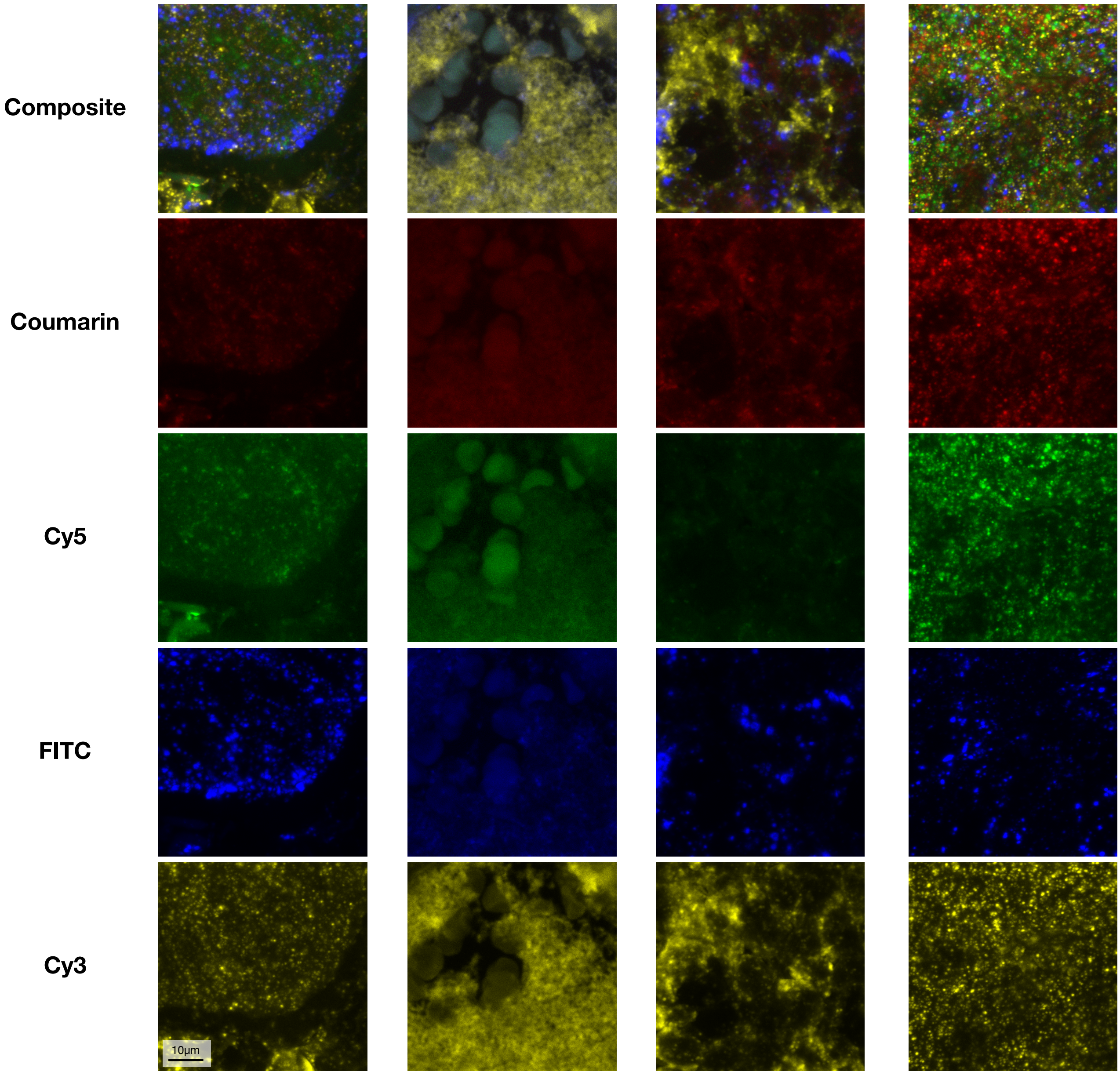

Supplementary Figure 7. Representative colocalisation images with single channels

Tissue array blocks were sectioned at 4μm and stained, multi-label, for FITC (488/517 nm, Blue), Cy3 (514/565 nm, Yellow), Cy5 (633/671 nm, Green), and Coumarin (405/470 nm, Red) for Cyclin D1, MMP7, c-Myc, and FRA1 respectively. Images were taken using a Leica SP8 confocal system at 40X magnification, 6X digital zoom. Samples were taken from a random set of tissue cores (n=12, 6 SCC and 6 NT).

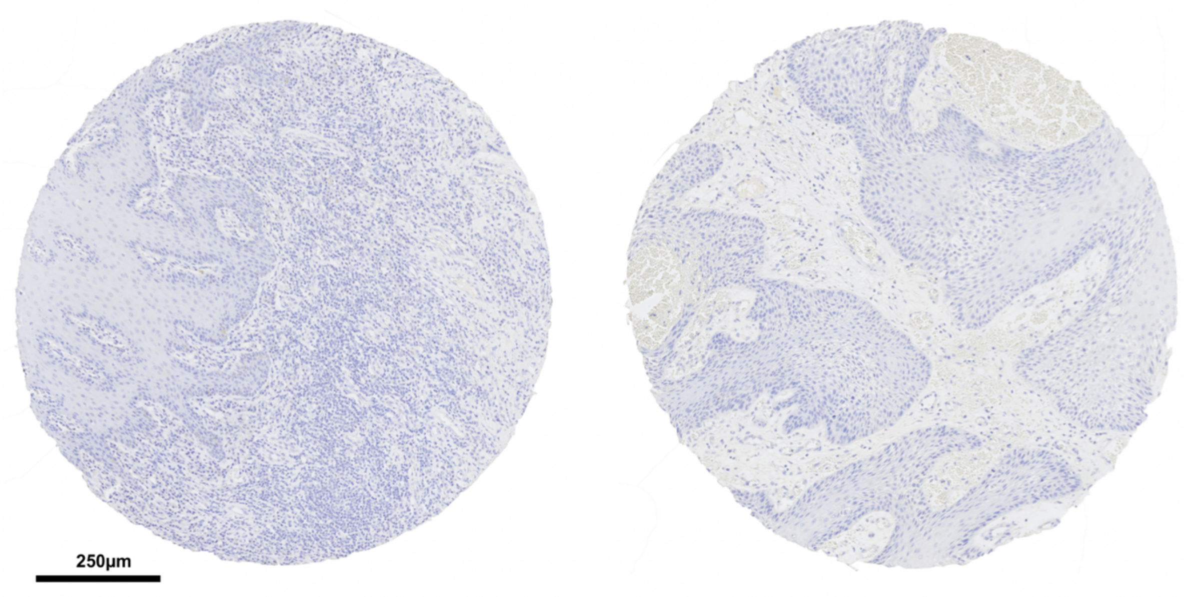

Supplementary Figure 8. No primary antibody controls.

A sister section (4µm) of the tissue array was stained immunohistochemically with no primary antibody. Slides were imaged at 40X magnification on a Hamamatsu slide scanner. No DAB stain can be seen in the control tissue slides.
